# Supplementary material for: Supply-side readiness to deliver HIV testing and treatment services in Indonesia: Going the last mile to eliminate mother-to-child transmission of HIV
Source: PLOS Glob Public Health. 2022 Aug 3;2(8):e0000845. doi: 10.1371/journal.pgph.0000845 (PMC10021386; doi:10.1371/journal.pgph.0000845)
Supplement: S1 Table — (DOCX) [file pgph.0000845.s001.docx]

| **S1 Table. List of variables for logistic regression model** | | |
| --- | --- | --- |
| **Variables** | **Definitions** | **Classifications** |
| ANC readiness score | The sum of indicators for ANC services divided by 8 (number of indicators) | Categorical variable with ANC readiness score of 0 to 0.65 coded as 0, 0.66 to 0.80 coded as 1 and 0.81 to 1 coded as 2 |
| PMTCT readiness score | The sum of indicators for PMTCT services divided by 14 (number of indicators) | Categorical variable with PMTCT readiness score of 0 to 0.22 coded as 0, 0.23 to 0.40 coded as 1 and 0.41 to 1 coded as 2 |
| HCS readiness score | The sum of indicators of HCS services divided by 9 (number of indicators) | Categorical variable with HCS readiness score of 0 to 0.35 coded as 0, 0.36 to 0.50 coded as 1 and 0.51 to 1 coded as 2 |
| Region | Classification of geographical location of health facilities in Indonesia | Categorical binary variable (i.e., Java-Bali and Outer Java-Bali) |
| Areas | Classification of health facility location, whether in urban or rural areas | Categorical binary variable (i.e., urban and rural) |
| Type of Service | Classification between health facilities that were equipped for Basic Emergency, Obstetric and Neonatal Care (BEONC) or not | Categorical binary variable (i.e., BEONC or non-BEONC) |
| Financial management | Classification based on the type of financial management in the facilities, where BLUD is for-profit and non-BLUD is not-for-profit | Categorical binary variable (i.e.,) BLUD or non-BLUD |
| Village midwives | Number of village midwives linked to the facility | Numerical variable |
| HIV counsellors | Number of trained HIV testing counsellors onsite | Numerical variable |
| Community health workers (CHWs) | Number community health workers in the health facilities | Numerical variable |
| “*Posyandu*” (community health post) | Number of community health posts by health facility in the area | Numerical variable |
