# Supplementary material for: Supply-side readiness to deliver HIV testing and treatment services in Indonesia: Going the last mile to eliminate mother-to-child transmission of HIV
Source: PLOS Glob Public Health. 2022 Aug 3;2(8):e0000845. doi: 10.1371/journal.pgph.0000845 (PMC10021386; doi:10.1371/journal.pgph.0000845)
Supplement: S2 Table — (DOCX) [file pgph.0000845.s002.docx]

| **S2 Table. Multivariate logistic regression model with 5% threshold** | | | | | | | | | | | | | |
| --- | --- | --- | --- | --- | --- | --- | --- | --- | --- | --- | --- | --- | --- |
|  | | **Model 1: ANC Readiness Score** | | | | **Model 2: PMTCT Readiness Score** | | | | **Model 3: HCS Readiness Score** | | | |
| **Variables** | | **uOR (95% CI)** | **p** | **aOR (95% CI)** | **p** | **uOR (95% CI)** | **p** | **aOR (95% CI)** | **p** | **uOR (95% CI)** | **p** | **aOR (95% CI)** | **P** |
| Readiness score^1^ | 0 | 1.00 |  | 1.00 |  | 1.00 |  | 1.00 |  | 1.00 |  | 1.00 |  |
|  | 1 | 2.77 (0.71 to 10.78) | 0.141 | 6.00 (0.55 to 65.02) | 0.140 | 5.25 (2.05 to 13.44)** | 0.001 | 0.81 (0.11 to 6.09) | 0.837 | 1.54 (0.50 to 4.69) | 0.450 | 2.32 (0.36 to 14.80) | 0.372 |
|  | 2 | 11.12 (3.48 to 35.55)** | <0.001 | 17.46 (2.86 to 106.45)** | 0.002 | 34.71 (6.92 to 174.06)** | <0.001 | 10.59 (1.51 to 74.25)** | 0.018 | 3.34 (1.16 to 9.62)** | 0.025 | 2.77 (0.38 to 20.02) | 0.314 |
| Region | Java-Bali | 1.00 |  | 1.00 |  | 1.00 |  | 1.00 |  | 1.00 |  | 1.00 |  |
|  | Outer Java-Bali | 0.007 (0.003 to 0.020)** | <0.001 | 0.04 (0.01 to 0.17)** | <0.001 | 0.007 (0.003 to 0.020)** | <0.001 | 0.02 (0.004 to 0.097)** | <0.001 | 0.007 (0.003 to 0.020)** | <0.001 | 0.03 (0.005 to 0.137)** | <0.001 |
| Areas | Urban | 1.00 |  | 1.00 |  | 1.00 |  | 1.00 |  | 1.00 |  | 1.00 |  |
|  | Rural | 0.11 (0.05 to 0.28)** | <0.001 | 0.55 (0.12 to 2.43) | 0.431 | 0.11 (0.05 to 0.28)** | <0.001 | 0.52 (0.07 to 3.58) | 0.504 | 0.11 (0.05 to 0.28)** | <0.001 | 0.71 (0.17 to 2.96) | 0.642 |
| Type of service | BEONC^2^ | 1.00 |  |  |  | 1.00 |  |  |  | 1.00 |  |  |  |
|  | Non-BEONC^2^ | 0.55 (0.23 to 1.33) | 0.185 |  |  | 0.55 (0.23 to 1.33) | 0.185 |  |  | 0.55 (0.23 to 1.33) | 0.185 |  |  |
| Type of Financial Management | BLUD^3^ | 1.00 |  |  |  | 1.00 |  |  |  | 1.00 |  |  |  |
|  | Non-BLUD^3^ | .99 (0.35 to 2.86) | 0.996 |  |  | 0.99 (0.35 to 2.86) | 0.996 |  |  | 0.99 (0.35 to 2.86) | 0.996 |  |  |
| Number of village midwives | | 0.87 (0.82 to 0.93)** | <0.001 | 0.96 (0.86 to 1.08) | 0.495 | 0.87 (0.82 to 0.93)** | <0.001 | 0.95 (0.83 to 1.09) | 0.508 | 0.87 (0.82 to 0.93)** | <0.001 | 0.95 (0.84 to 1.07) | 0.422 |
| Number of trained counsellors | | 3.37 (1.99 to 5.72)** | <0.001 | 1.95 (1.05 to 3.64)** | 0.033 | 3.37 (1.99 to 5.72)** | <0.001 | 1.96 (1.24 to 3.11)** | 0.004 | 3.37 (1.99 to 5.72)** | <0.001 | 1.82 (1.09 to 3.04)** | 0.023 |
| Number of CHWs | | 1.01 (1.01 to 1.02)** | <0.001 | 1.01 (1.00 to 1.01)** | 0.008 | 1.01 (1.01 to 1.02)** | <0.001 | 1.00 (0.99 to 1.01) | 0.104 | 1.01 (1.01 to 1.02)** | <0.001 | 1.01 (1.00 to 1.02)* | 0.091 |
| Number of community health post (“posyandu”) | | 1.05 (1.03 to 1.09)** | <0.001 | 1.00 (0.97 to 1.04) | 0.846 | 1.05 (1.03 to 1.09)** | <0.001 | 0.98 (0.94 to 1.02) | 0.381 | 1.05 (1.03 to 1.09)** | <0.001 | 0.98 (0.94 to 1.03) | 0.484 |
| **NOTES:**  **p<0.05; *P<0.1  ANC: antenatal care; aOR: adjusted odds ratio; CHWs: community health workers; CI: confidence interval; HCS: HIV care and support; PMTCT: Prevention Mother-to-Child Transmission; uOR: unadjusted odds ratio  ^1^Model 1 used ANC readiness scores, model 2 used PMTCT readiness scores and model 3 used HCS readiness scores  ^2^BEONC: Basic Emergency, Obstetric and Neonatal Care, referring to the health facilities (“*Puskesmas*”) that were equipped with the capacity to provide basic obstetric and neonatal emergency care  ^3^”*Badan Layanan Umum Daerah*/*BLUD*” is a term used for a public district organization that provides services to the community with the flexibility to implement business models to support revenue generation and to improve efficiency | | | | | | | | | | | | | |
