# Supplementary material for: Supply-side readiness to deliver HIV testing and treatment services in Indonesia: Going the last mile to eliminate mother-to-child transmission of HIV
Source: PLOS Glob Public Health. 2022 Aug 3;2(8):e0000845. doi: 10.1371/journal.pgph.0000845 (PMC10021386; doi:10.1371/journal.pgph.0000845)
Supplement: S3 Table — (DOCX) [file pgph.0000845.s003.docx]

| **S3 Table. Multivariate logistic regression model with 15% threshold** | | | | | | | | | | | | | |
| --- | --- | --- | --- | --- | --- | --- | --- | --- | --- | --- | --- | --- | --- |
|  | | **Model 1: ANC Readiness Score** | | | | **Model 2: PMTCT Readiness Score** | | | | **Model 3: HCS Readiness Score** | | | |
| **Variables** | | **uOR (95% CI)** | **p** | **aOR (95% CI)** | **p** | **uOR (95% CI)** | **p** | **aOR (95% CI)** | **p** | **uOR (95% CI)** | **p** | **aOR (95% CI)** | **P** |
| Readiness score^1^ | 0 | 1.00 |  | 1.00 |  | 1.00 |  | 1.00 |  | 1.00 |  | 1.00 |  |
|  | 1 | 2.89 (0.70 to 11.97) | 0.144 | 4.87 (0.63 to 37.68) | 0.129 | 5.28 (2.73 to 14.53)** | 0.001 | 1.96 (0.29 to 13.17) | 0.487 | 1.88 (0.54 to 6.54) | 0.319 | 2.60 (0.38 to 17.71) | 0.328 |
|  | 2 | 8.51 (2.49 to 29.08)** | 0.001 | 5.42 (1.08 to 27.27)** | 0.040 | 40.30 (8.82 to 184.12)** | <0.001 | 25.15 (4.29 to 159.54)** | <0.001 | 4.06 (1.25 to 13.15)** | 0.020 | 4.74 (0.75 to 30.02)* | 0.098 |
| Region | Java-Bali | 1.00 |  | 1.00 |  | 1.00 |  | 1.00 |  | 1.00 |  | 1.00 |  |
|  | Outer Java-Bali | 0.01 (0.003 to 0.026)** | <0.001 | 0.03 (0.003 to 0.243)** | 0.001 | 0.01 (0.003 to 0.026)** | <0.001 | 0.02 (0.002 to 0.112)** | <0.001 | 0.01 (0.003 to 0.026)** | <0.001 | 0.02 (0.002 to 0.150)** | <0.001 |
| Areas | Urban | 1.00 |  | 1.00 |  | 1.00 |  | 1.00 |  | 1.00 |  | 1.00 |  |
|  | Rural | 0.18 (0.07 to 0.45)** | <0.001 | 1.56 (0.29 to 8.41) | 0.607 | 0.18 (0.07 to 0.45)** | <0.001 | 2.06 (0.19 to 21.77) | 0.548 | 0.18 (0.07 to 0.45)** | <0.001 | 2.08 (0.49 to 8.90) | 0.323 |
| Type of service | BEONC^2^ | 1.00 |  |  |  | 1.00 |  |  |  | 1.00 |  |  |  |
|  | Non-BEONC^2^ | 0.51 (0.21 to 1.27) | 0.149 |  |  | 0.51 (0.21 to 1.27) | 0.149 |  |  | 0.51 (0.21 to 1.27) | 0.149 |  |  |
| Type of Financial Management | BLUD^3^ | 1.00 |  |  |  | 1.00 |  |  |  | 1.00 |  |  |  |
|  | Non-BLUD^3^ | 1.23 (0.42 to 3.55) | 0.703 |  |  | 1.23 (0.42 to 3.55) | 0.703 |  |  | 1.23 (0.42 to 3.55) | 0.703 |  |  |
| Number of village midwives | | 0.89 (0.84 to 0.95)** | <0.001 | 0.98 (0.86 to 1.13) | 0.839 | 0.89 (0.84 to 0.95)** | <0.001 | 0.99 (0.87 to 1.12) | 0.857 | 0.89 (0.84 to 0.95)** | <0.001 | 0.99 (0.86 to 1.13) | 0.853 |
| Number of trained counsellors | | 3.19 (1.93 to 5.26)** | <0.001 | 2.03 (1.21 to 3.40)** | 0.007 | 3.19 (1.93 to 5.26)** | <0.001 | 2.06 (1.27 to 3.33)** | 0.003 | 3.19 (1.93 to 5.26)** | <0.001 | 1.98 (0.86 to 1.13)** | 0.005 |
| Number of active health CHWs in the community | | 1.01 (1.01 to 1.02)** | <0.001 | 1.00 (0.95 to 1.05) | 0.966 | 1.01 (1.01 to 1.02)** | <0.001 | 0.98 (0.93 to 1.02) | 0.366 | 1.01 (1.01 to 1.02)** | <0.001 | 0.99 (0.94 to 1.04) | 0.655 |
| Number of community health post (“posyandu”) | | 1.05 (1.02 to 1.08)** | <0.001 | 1.00 (1.00 to 1.01)* | 0.053 | 1.05 (1.02 to 1.08)** | <0.001 | 1.00 (0.99 to 1.01) | 0.157 | 1.05 (1.02 to 1.08)** | <0.001 | 1.00 (1.00 to 1.01)* | 0.093 |
| **NOTES:**  **p<0.05; *P<0.1  ANC: antenatal care; aOR: adjusted odds ratio; CHWs: community health workers; CI: confidence interval; HCS: HIV care and support; PMTCT: Prevention Mother-to-Child Transmission; uOR: unadjusted odds ratio  ^1^Model 1 used ANC readiness scores, model 2 used PMTCT readiness scores and model 3 used HCS readiness scores  ^2^BEONC: Basic Emergency, Obstetric and Neonatal Care, referring to the health facilities (“*Puskesmas*”) that were equipped with the capacity to provide basic obstetric and neonatal emergency care  ^3^”*Badan Layanan Umum Daerah*/*BLUD*” is a term used for a public district organization that provides services to the community with the flexibility to implement business models to support revenue generation and to improve efficiency | | | | | | | | | | | | | |
