# Supplementary material for: Supply-side readiness to deliver HIV testing and treatment services in Indonesia: Going the last mile to eliminate mother-to-child transmission of HIV
Source: PLOS Glob Public Health. 2022 Aug 3;2(8):e0000845. doi: 10.1371/journal.pgph.0000845 (PMC10021386; doi:10.1371/journal.pgph.0000845)
Supplement: S4 Table — (DOCX) [file pgph.0000845.s004.docx]

| **S4 Table. Multivariate regression model with the rate of absolute change in antenatal HIV testing from 2016 to 2018** | | | | | |
| --- | --- | --- | --- | --- | --- |
| **Variables** | | **Coefficient (95% CI)** | **p** | **Coefficient (95% CI)** | p |
| ANC readiness score | 0 | 0.00 |  |  |  |
|  | 1 | -0.08 (-0.63 to 0.47) | 0.763 |  |  |
|  | 2 | -0.26 (-0.76 to 0.23) | 0.291 |  |  |
| PMTCT readiness score | 0 | 0.00 |  |  |  |
|  | 1 | 0.02 (-0.28 to 0.31) | 0.897 |  |  |
|  | 2 | -0.05 (-0.40 to 0.29) | 0.765 |  |  |
| HCS readiness score | 0 | 0.00 |  |  |  |
|  | 1 | 0.18 (-0.18 to 0.54) | 0.333 |  |  |
|  | 2 | 0.11 (-0.20 to 0.42) | 0.480 |  |  |
| **Region** | **Java-Bali** | **0.00** |  | 0.00 |  |
|  | **Non-Java-Bali** | **0.32 (0.03 to 0.62)** | **0.032** | 0.12 (-0.07 to 0.32) | 0.213 |
| Areas | Urban | 0.00 |  |  |  |
|  | Rural | -0.02 (-0.30 to 0.27) | 0.903 |  |  |
| Type of services | BEONC | 0.00 |  |  |  |
|  | Non-BEONC | -0.02 (-0.31 to 0.26) | 0.877 |  |  |
| **Type of Financial Managements** | **BLUD** | **0.00** |  | 0.00 |  |
|  | **Non-BLUD** | **-0.037 (-0.68 to -0.06)** | **0.018** | -0.07 (-0.02 to 0.02) | 0.412 |
| **Number of village midwives** | | **0.02 (0.002 to 0.049)** | **0.036** | -0.001 (-0.018 to 0.016) | 0.910 |
| Number of trained counsellors | | -0.08 (-0.17 to 0.02) | 0.116 |  |  |
| Number of active health cadres in the community | | -0.001 (-0.001 to 0.0002) | 0.122 |  |  |
| Number of community health posts | | 0.001 (-0.005 to 0.008) | 0.674 |  |  |
| **Outcome is a continuous variable of the rate of absolute change in antenatal HIV testing per facility from 2016 to 2018** | | | | | |
