# Supplementary material for: Supply-side readiness to deliver HIV testing and treatment services in Indonesia: Going the last mile to eliminate mother-to-child transmission of HIV
Source: PLOS Glob Public Health. 2022 Aug 3;2(8):e0000845. doi: 10.1371/journal.pgph.0000845 (PMC10021386; doi:10.1371/journal.pgph.0000845)
Supplement: S5 Table — (DOCX) [file pgph.0000845.s005.docx]

| **S5 Table. Modified PMTCT readiness score with one indicator excluded*** | | |
| --- | --- | --- |
| **Service area (total indicators)** | **Readiness indicator** | **Study facilities with indicators n (%)** |
| PMTCT (14 indicators) | Guidelines for PMTCT | 94 (16.9) |
|  | Guidelines for infants and young child feeding | 171 (30.7) |
|  | Guidelines for antiretroviral therapy | 8 (1.4) |
|  | Staff trained in PMTCT | 106 (19.0) |
|  | Staff trained in infant and young child feeding | 125 (22.4) |
|  | HIV rapid testing kit (HIV diagnostic capacity for adults) | 116 (20.8) |
|  | Zidovudine (AZT) | 5 (0.9) |
|  | Nevirapine (NVP) | 6 (1.1) |
|  | Maternal antiretroviral prophylaxis | 25 (4.5) |
|  | CD4 or viral load | 7 (1.3) |
|  | Renal function test | 69 (12.4) |
|  | Liver function test | 61 (10.9) |
|  | Three first line antiretroviral Option B+ (AZT+ NVP + 3TC) | 6 (1.1) |
| **Modified Cronbach alpha score for PMTCT (unstandardized)** | | **0.69** |
| **Modified Cronbach alpha score for PMTCT (standardized)** | | **0.75** |
| *One indicator that were excluded is the availability of room with visual and auditory privacy | | |
