# Supplementary material for: Supply-side readiness to deliver HIV testing and treatment services in Indonesia: Going the last mile to eliminate mother-to-child transmission of HIV
Source: PLOS Glob Public Health. 2022 Aug 3;2(8):e0000845. doi: 10.1371/journal.pgph.0000845 (PMC10021386; doi:10.1371/journal.pgph.0000845)
Supplement: S6 Table — (DOCX) [file pgph.0000845.s006.docx]

| **S6 Table. Results of multivariate logistic regression model of factors associated with the proportion of ≥10% of pregnant women tested for HIV per facility in public facilities, in 2016 in Indonesia^1^** | | | | | |
| --- | --- | --- | --- | --- | --- |
| **Variables** | | **uOR (95% CI)** | **p** | **aOR (95% CI)** | **p** |
| PMTCT readiness score | 0 | 1.00 |  | 1.00 |  |
|  | 1 | 8.0 (3.1 to 20.7) | <0.001** | 1.87 (0.45 to 7.78) | 0.386 |
|  | 2 | 138.7 (15.4 to 1250.7) | <0.001** | 10.85 (0.85 to 139.17) | 0.067* |
| Regions | Java-Bali | 1.00 |  | 1.00 |  |
|  | Outer Java-Bali | 0.03 (0.01 to 0.08) | <0.001** | 0.03 (0.01 to 0.14) | <0.001** |
| Areas | Urban | 1.00 |  | 1.00 |  |
|  | Rural | 0.14 (0.06 to 0.33) | <0.001** | 1.03 (0.21 to 5.11) | 0.972 |
| Type of services | BEONC | 1.00 |  |  |  |
|  | Non-BEONC^2^ | 0.73 (0.34 to 1.56) | 0.413 |  |  |
| Type of financial managements | BLUD | 1.00 |  | 1.00 |  |
|  | Non-BLUD^3^ | 0.34 (0.13 to 0.87) | 0.025** | 0.11 (0.02 to 0.73) | 0.022** |
| Number of village midwives | | 0.89 (0.84 to 0.95) | <0.001** | 0.98 (0.86 to 1.12) | 0.809 |
| Number of trained counsellors | | 2.3 (1.7 to 3.1) | <0.001** | 1.77 (1.16 to 2.69) | 0.008** |
| Number of active CHWs | | 1.01 (1.003 to 1.0011) | <0.001** | 0.99 (0.99 to 1.00) | 0.822 |
| Number of community health posts | | 1.04 (1.02 to 1.06) | <0.001** | 1.00 (0.96 to 1.04) | 0.916 |
| **NOTES:**  *p-value: <0.05; *p-value: <0.1  PMTCT readiness score: 0 = 0 – 0.22; 1 = 0.22 – 0.40  ^1^This model used a modified PMTCT readiness score available in S5 Table  ^2^BEONC: Basic Emergency, Obstetric and Neonatal Care, referring to the health facilities (“*Puskesmas*”) that were equipped with the capacity to provide basic obstetric and neonatal emergency care  ^3^”*Badan Layanan Umum Daerah*/*BLUD*” is a term used for a public district organization that provides services to the community with the flexibility to implement business models to support revenue generation and to improve efficiency | | | | | |
