# Supplementary material for: Supply-side readiness to deliver HIV testing and treatment services in Indonesia: Going the last mile to eliminate mother-to-child transmission of HIV
Source: PLOS Glob Public Health. 2022 Aug 3;2(8):e0000845. doi: 10.1371/journal.pgph.0000845 (PMC10021386; doi:10.1371/journal.pgph.0000845)
Supplement: Attachment — Submitted filename: Response to reviewer #2.docx [file pgph.0000845.s007.docx]

**Date: 1^st^ July 2022**

Re: Manuscript Title: Supply-side readiness to deliver HIV testing and treatment service in Indonesia: Going the last mile to eliminate mother-to-child transmission of HIV

**ID: PGPH-D-21-00735**

We are very appreciative of the comments and suggestions offered by the editor and reviewers. Their advice has strengthened the article. Below are our responses to their comments.

**Editor**

1. **Editor comment:** “Thank you for your patience. Please address comments from Reviewer #1, with emphasis on explanation of how private midwifes fit into your analysis. In addition, there are several issues with the model results presented in Table 2 that should be addressed.”

**Response:** Thank you. Please find below the details of revisions made based on your valuable comments.

2. **Editor comment:** “The Readiness Scores presented in Table 2 do not seem to align with the explanation provided in the Methods whereby each score should be a proportion. Please explain the definition and meaning of the 0, 1, 2 readiness scores presented in Table 2 in the Methods and in a footnote for Table 2.”

**Response:** Thank you for pointing this out. The definition for our readiness score can be found in Supplementary Table 1 (Supplementary Materials). A sentence about the categorical classification for the readiness scores has been added to the Methods section (see “Statistical analysis”, page 9). Table 2 also includes a footnote that defines these categories.

**Revised version: *“****Univariate and multivariate regressions were used to explore the relationship between the explanatory variables and the uptake of HIV testing by pregnant women across three types of services (ANC, PMTCT, and HCS) in public health facilities. For regression models, ANC, PMTCT and HCS the readiness scores were transformed into categorical variables: for ANC, 0 referred to an ANC readiness score of 0 to 0.65, 1 referred to 0.66 to 0.80 and 2 referred to 0.81 to 1; for PMTCT, 0 referred to PMTCT readiness score of 0 to 0.22, 1 referred to 0.23 to 0.40 and 2 referred to 0.41 to 1; and for HCS, 0 referred to HCS readiness score of 0 to 0.35, 1 referred to 0.36 to 0.50 and 2 referred to 0.51 to 1.”*

3. **Editor comment:** “In the description of Table 2 in the Results (page 14), higher readiness score is associated with the outcome in univariate analysis. As these are the primary exposure variables, the associations for these variables in the multivariable model should also be described in Results.”

**Response:** The sentence in the Results section (page 15) has been revised to also describe the relationship between the readiness score and the outcome.

**Revised version*: “****The multivariate analysis shows that the proportion of pregnant women tested for HIV was more likely to be greater than or equal to 10% in facilities with a higher readiness score, a higher number of trained counsellors available, and less likely in facilities located outside of Java-Bali or in facilities supporting a higher number of village midwives.”*

4. **Editor comment:** “Page 9 in Methods describes the outcome variable as HIV testing among pregnant women >= 10%. Why was this threshold used? If it’s a standard threshold, please explain and provide citation.”

**Response:** A threshold of 10% is used to represent the national antenatal HIV testing uptake rate in 2016 in Indonesia. This is explained on page 7 under the sub-heading “Proportion of pregnant women tested for HIV per facility”. We have attempted to make this clearer in the revised version as stated below.

**Revised version:** “*The primary outcome was defined as testing for HIV among pregnant women per facility greater than or equal to 10% (≥10%) – this cut-off represented the national testing coverage for HIV among pregnant women in Indonesia in 2016* (1)*.”*

5. **Editor comment:** “Please explain in the Methods why you chose to fit three separate models for each readiness score exposure variable, instead of including all three in one model.”

**Response:** In Indonesia, HIV testing and treatment are key components of the prevention of mother-to-child transmission (PMTCT). The PMTCT program is a cascade of care that includes stepwise activities beginning with all pregnant women attending their first antenatal care (ANC) visit followed by subsequent care. Our three separate models represent different steps in the PMTCT cascade of care. A few sentences have been added to the Methods section on page 8 and 9 to make this clearer.

**Revised version: *“****Since this is an ecological study using data collected at the health facility level, supply-side readiness will from hereon be referred to as ‘health facility readiness’. The health facility readiness scores were calculated for ANC, PMTCT, and HIV care and support services (HCS). These three readiness scores (i.e., for ANC, PMTCT and HCS) represent the different steps in the PMTCT program cascade of care.”*

***“****The composite readiness score represents the sum of total service readiness scores divided by the total number of indicators. The composite readiness score is calculated for ANC, PMTCT, and HCS to provide descriptive information on health facility readiness for each cascade of care along with the PMTCT program.”*

6. **Editor comment:** “In the Introduction of the Abstract, it would be helpful to briefly define readiness.”

**Response:** A sentence briefly defining ‘readiness’ has been added to the Abstract.

**Revised version:** *“Supply-side readiness scores - generated from a readiness index that measures overall structural capacity and is often used as proxy for quality of care - were adapted from the WHO Service Availability and Readiness Assessment Tool and presented by sector and geographic area.”*

7. **Editor comment:** “In the Abstract, multivariable analysis is mentioned in the Methods, but the Results section does not include findings from the multivariable analysis. Please add a brief summary of the findings in Results.”

**Response:** Thank you for pointing this out. Results from the multivariable analysis have now been added to the Abstract.

**Revised version:** “*The national composite readiness score for PMTCT was only 0.13 (based on a maximum score of 1) with a composite score of 0.21 for public facilities and 0.06 for private facilities. The multivariate analysis shows that the proportion of pregnant women tested for HIV was more likely to be greater than or equal to 10% in facilities with a higher readiness score and a higher number of trained counsellors available, and less likely in facilities located outside of Java-Bali and in facilities supporting a higher number of village midwives.”*

8. **Editor comment:** “Page 9. Cronbach’s alpha for PMTCT readiness scores is low at 0.630. Please describe which items are responsible for the low alpha. A sensitivity analysis with a modified PMTCT readiness score with the items removed might be useful.”

**Response:** The low Cronbach’s alpha for PMTCT is mostly caused by one indicator on visual and auditory privacy since it was consistently low in public facilities while it was high in most private facilities. The initial Cronbach alpha for PMTCT is 0.63 (unstandardized) and 0.73 (standardized). With this indicator (i.e., visual and auditory privacy) excluded from the scores, Cronbach’s alpha is increased to 0.69 (unstandardized) and 0.75 (standardized). Supplementary materials now includes new modified PMTCT readiness scores (with one indicator removed) and results of a sensitivity analysis using the threshold of 10% and the new modified score (Supplementary Table 4 and 5). A sentence is also added on page 10 to provide information about this additional supplementary analysis.

**Revised version: *“****Sensitivity analysis was performed to test the impact of changing the threshold for the proportion of pregnant women tested for HIV per facility to 5% and 15% and to test the impact of using modified PMTCT readiness scores (sensitivity analyses are available as Supplementary Materials).”*

9. **Editor comment:** “In page 11, the readiness scores for each three types of services are compared. Since the number of items and the content of the measures are different, it seems inappropriate to compare the scores across these different services. Please explain in Methods why this comparison is appropriate or remove the comparison from the paper.”

**Response:** Thank you for pointing this out. We removed the comparison made previously in page 12 to address this comment.

10. **Editor comment:** “References 31 & 34 are duplicates. Please eliminate one of them.”

**Response:** Thank you for pointing this out. Reference 34 has been removed from the Reference list.

11. **Editor comment:** “Some of the references seem to be missing information, including journal name, date, pages. For example, references 51 and 59, Please go through all of the references and make sure they have complete information in the correct format.”

**Response:** Thank you. The reference list has now been reviewed and any missing information provided.

**Reviewer #1**

1. **Reviewer’s comment:** “The description of the health system and MCH offer is quite complex, maybe a figure/map would help.”

**Response:** Thank you for this comment. A figure has been added under the Setting subsection on page 6.

**Revised version:**

**Figure 1.** Organization of health system in Indonesia

2. **Reviewer’s comment:** “Authors explain that many/most pregnant women seek care from private midwife clinics, but also explain that village midwives provide most MHC care – are these based in the private clinics? What are their links? The study sample includes private facilities, but does it include these private midwife clinics?”

**Response:** The village midwives’ program began in 1984 and was designed ensure a rural midwife was available in every village and to reduce persistently high rates of maternal mortality. This program involves 1 extra year of training (on top of the standard 3-year training for hospital midwives) to enable them to act as multi-purpose health providers with a focus on pregnancy, delivery, and post-partum care (2). Some midwives are employed by the central or local government, however, most of them have multiple sources of income, with an estimated 58% of their annual total income coming from private practices (2). The study sample of Quality Service and Delivery Survey (QSDS) 2016 - used in this study- includes private maternal health providers and private clinics (3). A sentence has been added to Materials and method section on page 5 in the revised document to provide more context.

**Revised version:** *“In 2013, more than 52% of pregnant women sought care at private midwife clinics, 16.6% at “puskesmas”, 10% at hospital, 10% at “posyandu”, 6% at “poskesdes” and 4.3% at private obstetrician/general practitioner clinics* (4)*. Village midwives are the dominant provider of maternal health care and have been dramatically scaled up by the Government of Indonesia since 1984* (5)*. Some village midwives are employed by the central or local government, however, most of them have multiple sources of income, with an estimated 58% of their annual total income coming from private practices (2).”*

3. **Reviewer comment**: “Is there a readiness threshold? above which the components of the health system are considered as "ready"?”

**Response:** Health facility readiness in this study is based on the WHO Service Availability and Readiness Assessment (SARA) which aims to measure the capacity of health facilities to deliver health services. Service readiness (or health facility readiness in this study context) is a prerequisite to the delivery of quality health care (6). According to the WHO SARA manual (7), there is no specific readiness threshold, the readiness indices are calculated for benchmarking and capturing the overall capacity that should be available in all facilities. Despite there being no pre-defined threshold, this metric is important to indicate the capacity of facilities to provide specific services as well as identify within- and between- countries' differences (6). A sentence has been added under Explanatory variables section on page 8 to provide more information.

**Revised version:** *“These indicators reflect the ability of a health care facility to provide specific services including staff and training, equipment, diagnostics, medicines and commodities* (8,9)*. There is no specific readiness threshold, however, this metric is important to indicate the capacity of facilities to provide specific services as well as identify within- and between- countries' differences* (6)*.”*

4. **Reviewer comment:** “The Indonesia PMTCT policy context seems to be specific. The integration of HIV testing in routine ANC in 2017 seems late compared to other countries (opt-out approach to testing, PICT integrated in mist African countries since early/mid 2000s). What are the reasons for that? Time between policy, political decision, funding decision and intervention implementation can be "quite long", this should be mentioned in the discussion to properly interpret findings”

**Response:** Thank you. In the Introduction, we explained that Indonesia adopted PMTCT as a national policy in 2005 using a voluntary counselling and testing approach and that Indonesia lagged behind neighbouring countries such as Vietnam (10). In the early implementation stages, it was observed that pregnant women have to be motivated to undertake tests and that the subsequent implementation of antiretrovirals provision was inconsistent (10). Only in 2017, following the Ministry of Health Regulation No 52/2017 (11), were the PMTCT guidelines updated to include HIV, syphilis, and hepatitis B as part of the minimum services provided to all pregnant women (1). A sentence has been added to the Discussion section to provide additional context around the development of Indonesia’s PMTCT policy.

**Revised version: “***Despite the Government of Indonesia’s commitment to the elimination of mother-to-child transmission of HIV, syphilis, and hepatitis B by 2022, testing coverage for HIV remains low and subsequent treatment uptake even lower. While the national PMTCT policy was adopted in 2005 in Indonesia using a voluntary counselling and testing approach, it was only in 2017 that the national guidelines introduced HIV testing as a part of routine testing in antenatal care settings* (1,10)*.”*

**Additional comments as part of Journal Requirement**

1. In your ethics statement in the Methods section and in the online submission form, please confirm whether all data were fully anonymized before you accessed them.

**Response:** Yes, I confirm that all data were fully anonymized before I accessed them. I add this statement in Methods section under ethical approval on page 10.

2. Please update the 'Competing Interests' statement in the system, including any COIs declared by your co-authors. If you have no competing interests to declare, please state "The authors have declared that no competing interests exist".

**Response:** The authors have declared that no competing interests exist. I add ‘Competing Interests’ statement in the end of the text on page 20.

3. Please amend your detailed Financial Disclosure statement. This is published with the article. It must therefore be completed in full sentences and contain the exact wording you wish to be published.

- State what role the funders took in the study. If the funders had no role in your study, please state: “The funders had no role in study design, data collection and analysis, decision to publish, or preparation of the manuscript.”

**Response:** The funders had no role in study design, data collection and analysis, decision to publish, or preparation of the manuscript. I add this statement under Funding section in the revised document on page 20.

4. Please provide separate figure files in .tif or .eps format and remove them from the manuscript file.

For more information about figure files please see our guidelines:

https://journals.plos.org/globalpublichealth/s/figures

https://journals.plos.org/globalpublichealth/s/figures#loc-file-requirements

**Response:** Figures that are used in the manuscript has been saved in .tif or .eps format and they are removed from the manuscript files (Figure 1, 2, 3 and 4).

5. We have noticed that you have uploaded Supporting Information files, but you have not included a list of legends. Please add a full list of legends for your Supporting Information files after the references list.

**Response:** Thank you for pointing this out. A full list of legends for our supplementary materials is added to the revised manuscript after the references list.

**References**

1.	World Health Organization. Review of the national health sector response to HIV in the republic of Indonesia [Internet]. 2017. Available from: https://www.who.int/docs/default-source/searo/indonesia/hiv-country-review-indonesia2017-eng.pdf?sfvrsn=fb6566d3_2

2.	Ensor T, Quayyum Z, Nadjib M, Sucahya P. Level and determinants of incentives for village midwives in Indonesia. Health Policy Plan [Internet]. 2009 Jan 1 [cited 2020 Aug 26];24(1):26–35. Available from: https://academic.oup.com/heapol/article/24/1/26/599250

3.	The World Bank. Is Indonesia Ready to Serve? [Internet]. 2018. Available from: https://documents.worldbank.org/en/publication/documents-reports/documentdetail/484351538653658243/is-indonesia-ready-to-serve-an-analysis-of-indonesia-s-primary-health-care-supply-side-readiness

4.	Wulandari LPL, Lubis DSM, Widarini P, Widyanthini DN, Wirawan IMA, Wirawan DN. HIV testing uptake among pregnant women who attending private midwife clinics: challenges of scaling up universal HIV testing at the private sectors in Indonesia. Int J Health Plann Manage. 2019 May 23;1–9.

5.	Kusuma D, Cohen J, Mcconnell M, Berman P. Can cash transfers improve determinants of maternal mortality? Evidence from the household and community programs in Indonesia. Soc Sci Med. 2016;163:10–20.

6.	Leslie HH, Spiegelman D, Zhou X, Kruk ME. Service readiness of health facilities in Bangladesh, Haiti, Kenya, Malawi, Namibia, Nepal, Rwanda, Senegal, Uganda and the United Republic of Tanzania. Bull World Health Organ [Internet]. 2017 [cited 2018 Jul 15];95(11):738–48. Available from: https://www.ncbi.nlm.nih.gov/pmc/articles/PMC5677617/pdf/BLT.17.191916.pdf

7.	World Health Organization. Service Availability and Readiness Assessment (SARA) An annual monitoring system for service delivery. 2015.

8.	World Health Organization. Service availability and readiness assessment (SARA). WHO [Internet]. 2019 [cited 2020 Aug 14]; Available from: http://www.who.int/healthinfo/systems/sara_introduction/en/

9.	Lipira L, Kemp C, Domercant JW, Honoré JG, Francois K, Puttkammer N. The role of service readiness and health care facility factors in attrition from option b+ in haiti: A joint examination of electronic medical records and service provision assessment survey data. Int Health [Internet]. 2018 [cited 2021 Jun 23];10(1):54–62. Available from: https://academic.oup.com/inthealth/article/10/1/54/4797301

10.	Hardon AP, Oosterhoff P, Imelda JD, Anh NT, Hidayana I. Preventing mother-to-child transmission of HIV in Vietnam and Indonesia: Diverging care dynamics. Soc Sci Med. 2009;69(6):838–45.

11.	Ministry of Health. Regulation of Minister of Health No 52 Year 2017 Republic of Indonesia on Elimination Mother-to-Child Transmission of HIV and Syphilis. 2017.
